# Anatomy of the nerves, vessels, and muscular compartments of the forearm, as revealed by high-resolution ultrasound. Part 2: vascularization of compartments and cutaneous innervation

**DOI:** 10.1590/0100-3984.2021.0031

**Published:** 2021

**Authors:** Áurea V. R. Mohana Borges, Sergio A. L Souza

**Affiliations:** 1 Department of Radiology, School of Medicine, Universidade Federal do Rio de Janeiro (UFRJ), Rio de Janeiro, RJ, Brazil.

**Keywords:** Ultrasonography/methods, Radial artery/anatomy & histology, Ulnar artery/anatomy & histology, Skin/innervation, Forearm/anatomy & histology

## Abstract

In recent decades, high-resolution ultrasound (HRUS) has revolutionized the morphological and structural evaluation of peripheral nerves and muscles, revealing details of the internal structure of the neural fascicles and muscle architecture. Applications range from diagnostics to interventional procedures. The anatomy of the forearm region is complex, with several muscles and an extensive network of vessels and nerves. To guarantee the success of the evaluation by HRUS, knowledge of the normal anatomy of the region is essential. The aim of these two companion articles is to present the normal anatomy of the nerves and compartments of the forearm, as revealed by HRUS, as well as the relationships between the main vessels and nerves of the region. Part 1 aims to review the overall structure of nerves, muscles and tendons, as seen on HRUS, and that of the forearm compartments. We present a practical approach, with general guidelines and tips on how best to perform the study. Part 2 is a pictorial essay about compartment vascularization and cutaneous innervation. The relationships between arteries, satellite veins and nerves, as well as the relationship between cutaneous nerves and superficial veins, are demonstrated. Knowledge of the normal anatomy of the forearm improves the technical quality of the examinations, contributing to better diagnoses, as well as improving the performance and safety of interventional procedures.

## INTRODUCTION

In recent decades, high-resolution ultrasound (HRUS) has revolutionized the morphological and structural evaluation of peripheral nerves and muscles^**(1)**^. To guarantee the success of the HRUS evaluation, knowledge of the normal anatomy of the region under study is essential. Familiarity with the relationships between the vessels and nerves in the forearm is important to ensure safety in interventional procedures.

The aim of these two companion articles is to present the normal anatomy of the nerves and compartments of the forearm, as revealed by HRUS, as well as the relationships between the main vessels and nerves of the region. Part 1 aims to review the overall structure of nerves, muscles and tendons, as seen on HRUS, and that of the forearm compartments. We present a practical approach, with general guidelines and tips on how best to perform the study. Part 2 is a pictorial essay about compartment vascularization and cutaneous innervation. In this second part, we focus on the relationships among arteries, satellite veins, and nerves, as well as on those among cutaneous nerves and superficial veins. The article is illustrated with images obtained with a high-resolution broadband 18-5 MHz linear transducer. All individuals depicted in the images were volunteers and gave written informed consent.

## VASCULARIZATION OF COMPARTMENTS AND RELATIONSHIPS BETWEEN VESSELS AND NERVES

### Arteries and nerves

**Figure 1:**
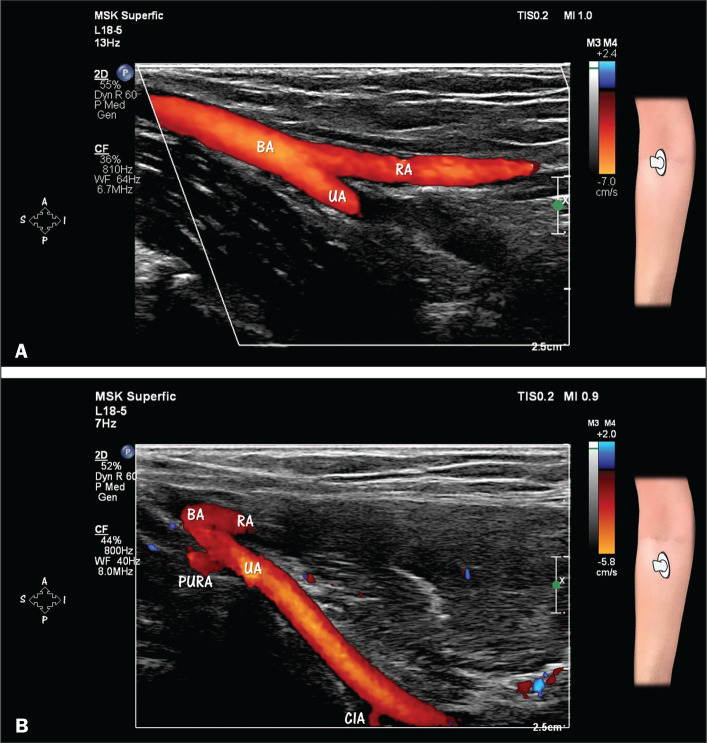
HRUS with Doppler, showing the bifurcation of the brachial artery (BA) into the ulnar artery (UA) and radial artery (RA). **A**: Note the more direct path of the RA as a distal continuation of the BA. **B**: The UA is in a deeper position than is the RA. Observe the origins of the posterior ulnar recurrent artery (PURA) and common interosseous artery (CIA) from the proximal part of the UA. The PURA is one of the two recurrent ulnar arteries and ascends in the posterior medial aspect of the elbow to anastomose with the superior ulnar collateral artery from the brachial artery. The CIA is a short vessel that bifurcates into the anterior and posterior interosseous arteries.

There are several neurovascular bundles in the forearm region, the vessels and nerves showing greater or lesser relative proximity along their course. The arterial blood supply is provided by the ulnar and radial arteries and their respective branches (Figure 1). When present, the median artery also supplies blood flow to the forearm.

**Figure 2:**
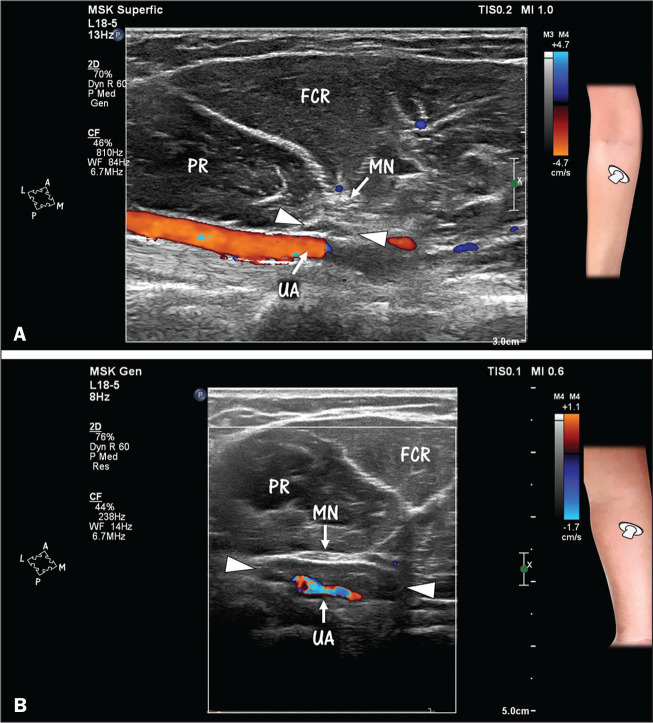
A,B: Separation of the initial part of the ulnar artery (UA) and the median nerve (MN) by the ulnar head of the pronator teres muscle (arrowheads) in the upper forearm. The UA is in a deep position, and the MN in a more superficial position. B: Note UA and MN compression caused by hypertrophy of the ulnar head of the pronator teres muscle (arrows). Hypertrophy of the ulnar head of the pronator teres muscle may be a cause of pronator syndrome. Both images were obtained by HRUS with Doppler. FCR, flexor carpi radialis (muscle); PR, pronator teres (muscle).

**Figure 3:**
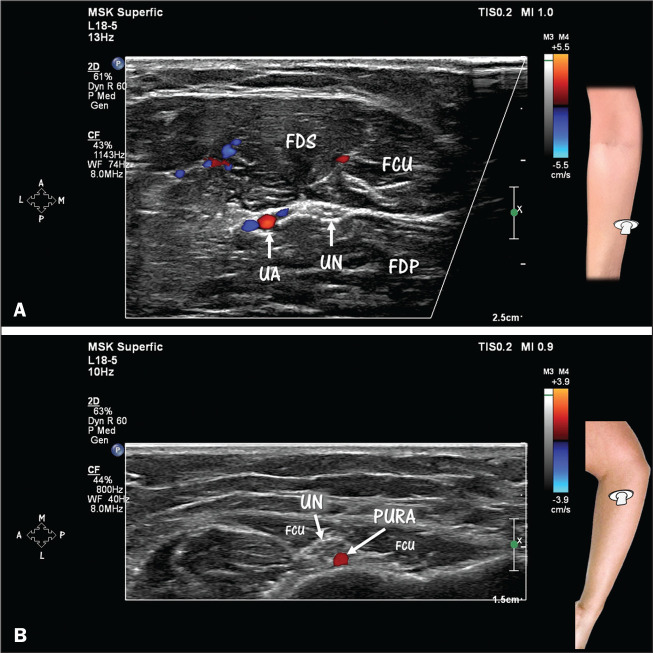
HRUS with Doppler, showing the anatomical relationships among the ulnar artery (UA), posterior ulnar recurrent artery (PURA), and ulnar nerve (UN). **A**: In the distal half of the forearm, the UA is in close proximity and lateral to the UN. Note the venae comitantes (in blue) near the UA (in red). The UN is visualized below the flexor carpi ulnaris (FCU) muscle. FDP, flexor digitorum profundus (muscle); FDS, flexor digitorum superficialis (muscle). **B**: In the most proximal portion of the forearm, note the relationship between the UN and the PURA, both of which are located between the two heads of the FCU muscle, the PURA being situated posterior to the UN.

The brachial artery divides into the ulnar and radial arteries at level of the elbow (Figure 1). The ulnar artery is typically the larger of the two and has an initial course that is more oblique than is that of the radial artery^**(2)**^. In its initial course, the ulnar artery passes below the ulnar head of the pronator teres muscle, which separates it from the median nerve (Figure 2). The median nerve, in turn, passes above the ulnar head of the muscle^**(3)**^. The ulnar artery and median nerve can be compressed by the ulnar head of the pronator muscle, which is one of the causes of pronator syndrome. The ulnar artery then passes between the flexor digitorum superficialis and flexor digitorum profundus muscles to the meeting point with the ulnar nerve in the distal half of the forearm^**(3)**^, as can be seen in Figure 3. The artery, satellite veins (venae comitantes), and nerve course towards the wrist among the flexor digitorum superficialis, flexor digitorum profundus, and flexor carpi ulnaris muscles. The ulnar artery is located laterally to the nerve. On HRUS, the ulnar nerve is visualized below the flexor carpi ulnaris muscle. In the most proximal portion of the forearm and inside the cubital tunnel, the posterior ulnar recurrent artery is in close proximity and posterior to the ulnar nerve^**(4)**^, as illustrated in Figure 3.

**Figure 4:**
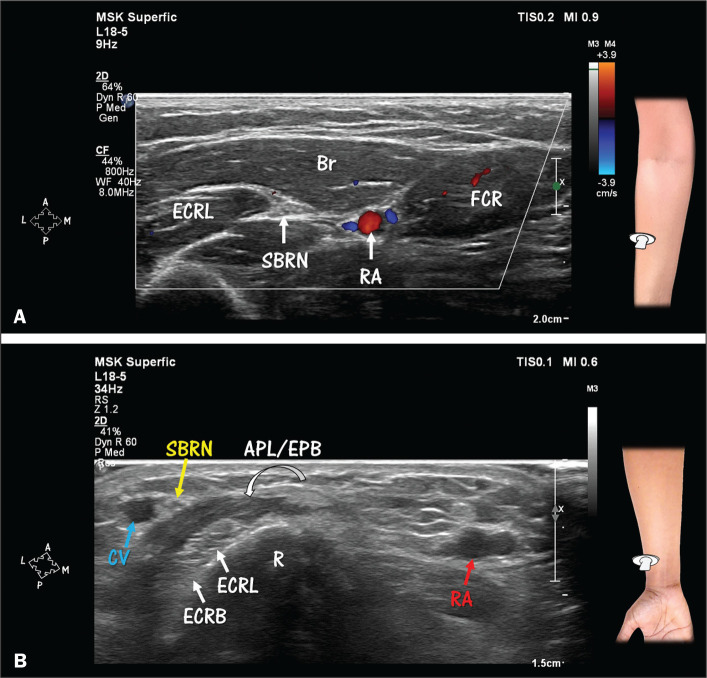
Anatomical relationship between the radial artery (RA) and the superficial branch of the radial nerve (SBRN). **A**: The RA is located medially to the SBRN. Note the venae comitantes (in blue) near the RA (in red) on HRUS with Doppler. The SBRN and RA are in closest proximity in the middle third of the forearm. Br, brachioradialis (muscle); ECRL, extensor carpi radialis longus (muscle); FCR, flexor carpi radialis (muscle). **B**: In the distal third of the forearm, there is relative separation of the RA from the SBRN. Note that the SBRN is in close proximity to the cephalic vein (CV) in the lateral portion of the dorsal compartment of the forearm. Nearby tendons of the abductor pollicis longus and extensor pollicis brevis (APL/EPB) muscles cross over the tendons of the ECRL and extensor carpi radialis brevis (ECRB) muscles (intersection). R, radius.

As depicted in Figure 1, the radial artery is usually smaller than the ulnar artery and is a more direct terminal branch of the brachial artery^**(2)**^. The radial artery crosses the forearm between the brachioradialis muscle on the lateral side and between the pronator teres and flexor carpi radialis muscles on the medial side. The superficial branch of the radial nerve is located near the radial artery, their proximity being greatest in the middle third of the forearm^**(3)**^, as shown in Figure 4. In the distal third of the forearm, there is a relative separation of the radial artery from the superficial branch of the radial nerve (Figure 4). The radial artery continues on an anterolateral course and the superficial branch of the radial nerve turns dorsally, piercing the fascia between the tendons of the brachioradialis and extensor carpi radialis longus muscles.

**Figure 5:**
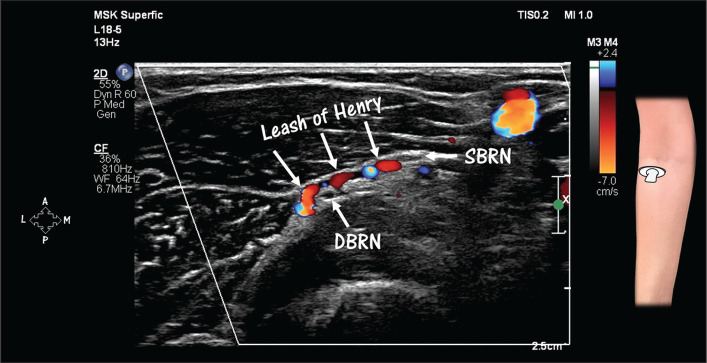
HRUS with Doppler, showing the leash of Henry, which corresponds to the transverse muscle branches of the radial recurrent artery and respective veins. These vessels supply the muscles of the lateral compartment and the supinator muscle. Note the vessels crossing between the deep branch (DBRN) and the superficial branch (SBRN) of the radial nerve at the level of the head of the radius.

The recurrent radial artery is a branch of the radial artery that originates in the proximal portion of the forearm^**(5)**^. The transverse branches of the vessel irrigate the brachioradialis, extensor carpi radialis longus, and extensor carpi radialis brevis muscles (lateral compartment), as well as the supinator muscle, and, together with the venae comitantes, form the so-called leash of Henry^**(6)**^, which is in close proximity with the deep and superficial branches of the radial nerve and can compress those structures (Figure 5).

**Figure 6:**
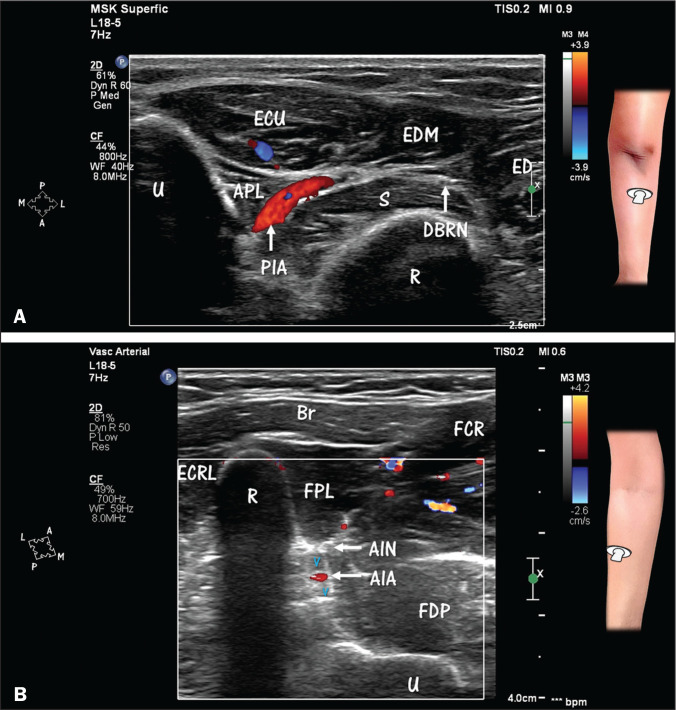
HRUS with Doppler of the forearm, showing the posterior and anterior interosseous blood vessels, as well as the nerves in the posterior compartment. **A**: Posterior interosseous artery (PIA) in its course between the supinator (S) muscle and the abductor pollicis longus (APL) muscle. Note the deep branch of the radial nerve (DBRN) in the distal portion of the supinator tunnel, from which it will emerge as the posterior interosseous nerve (posterior view). ECU, extensor carpi ulnaris (muscle); EDM, extensor digiti minimi (muscle); ED, extensor digitorum (muscle); U, ulna; R, radius. **B**: Anterior interosseous artery (AIA) and venae comitantes near the anterior surface of the interosseous membrane. Note the relationship between the vessels and the anterior interosseous nerve (AIN). Doppler evaluation facilitates the identification of the artery in relation to adjacent structures. Deep-seated veins have a very slow flow, which, under normal circumstances, makes it difficult to detect them with Doppler. Br, brachioradialis (muscle); FCR, flexor carpi radialis (muscle); ECRL, extensor carpi radialis longus (muscle); R, radius; FPL, flexor pollicis longus (muscle); FDP, flexor digitorum profundus (muscle); U, ulna.

The posterior interosseous artery originates from the common interosseous artery, one of the branches of the ulnar artery^**(4)**^. The posterior interosseous artery enters the posterior compartment above the interosseous membrane, emerging in the posterior compartment between the supinator and abductor pollicis longus muscles^**(7)**^, as illustrated in Figure 6. Upon reaching the intermuscular plane, between the superficial and deep layers, the posterior interosseous artery comes into closer proximity with the posterior interosseous nerve, after its emergence from the supinator tunnel^**(2,7)**^.

The anterior interosseous nerve accompanies the anterior interosseous artery along its course near the interosseous membrane. The artery, venae comitantes, and nerve cross the forearm between the edges of the flexor pollicis longus and flexor digitorum profundus muscles, after the emergence of anterior interosseous artery from the common interosseous artery^**(2)**^, as can be seen in Figures 1 and 6.

**Figure 7:**
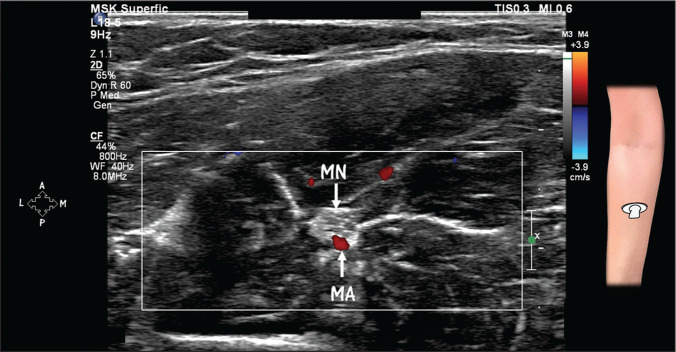
HRUS with Doppler, showing a persistent median artery (MA). Note the close relationship between the MA and the median nerve (MN).

The median artery, in turn, is a fetal artery that normally regresses as the radial and ulnar arteries develop. In approximately 30% of individuals, it persists into adulthood, following the course of the median nerve^**(8)**^. There are two major phenotypes of persistent median artery: palmar and antebrachial. In the palmar phenotype, the median artery continues to the level of the hand, whereas it does not extend past the forearm in the antebrachial phenotype (Figure 7).

### Cutaneous innervation

**Figure 8:**
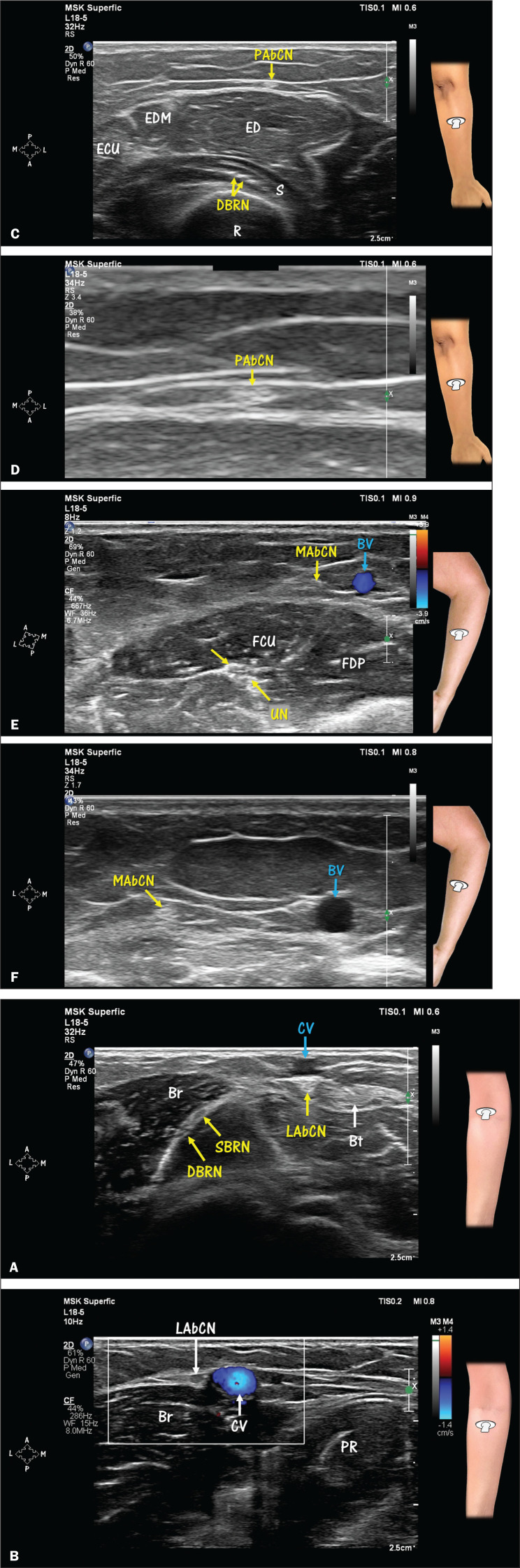
Cutaneous innervation of the forearm. **A,B**: Lateral antebrachial cutaneous nerve (LAbCN). The LAbCN is the terminal branch of the musculocutaneous nerve and provides sensory innervation to the lateral aspect of the forearm. **A**: At the level of the elbow, the LAbCN is identified in the lateral margin of the biceps tendon (Bt) and inferior to (deeper than) the cephalic vein (CV). At that same level, note the division of the radial nerve into the deep branch (DBRN) and superficial branch (SBRN). **B**: Note the close relationship between the LAbCN and the CV in the proximal portion of the forearm. This relationship makes the nerve susceptible to venipuncture-induced injury. The CV (in blue) can be distinguished from other structures by Doppler. Another way to identify the CV is to apply pressure with the transducer and note the collapse of the wall and its return to normal after decompression. Br, brachioradialis (muscle); PR, pronator teres (muscle). **C,D**: Posterior antebrachial cutaneous nerve (PAbCN). The PAbCN is a branch of the radial nerve that originates in the arm near the outlet of the spiral groove and provides sensory innervation to the posterior aspect of the forearm. Along the forearm, the nerve will be found above the extensor digitorum (ED) muscle as in C. Note the position of the nerve in the deep subcutaneous layer near the fascia. Also note (in C) fascicles of the deep branch of the radial nerve (DBRN) at a deeper position between the superficial and deep heads of the supinator (S) muscle, surrounded by echogenic fat. D: Zoom view of the PAbCN (magnification, ×3.4). EDM, extensor digiti minimi (muscle); ECU, extensor carpi ulnaris (muscle); R, radius. **E,F**: Medial antebrachial cutaneous nerve (MAbCN). The MAbCN is a direct branch of the brachial plexus and provides sensory innervation to the medial aspect of the forearm. Note the proximity of the nerve to the basilic vein (BV). E: The BV (in blue) can be distinguished from other structures by Doppler. F: Zoom view of the MAbCN and BV (magnification, ×1.7). FCU, flexor carpi ulnaris (muscle); FDP, flexor digitorum profundus (muscle); UN, ulnar nerve.

As depicted in Figure 8, cutaneous sensory innervation of the forearm is mainly supplied by three nerves^**(9)**^: the lateral antebrachial cutaneous nerve, a terminal branch of the musculocutaneous nerve; the posterior antebrachial cutaneous nerve, a branch of the radial nerve with origin in the arm; and the medial antebrachial cutaneous nerve, a direct branch of the brachial plexus.

### Veins and nerves

The main veins of the region are classified as superficial or deep veins. The superficial veins of the forearm are the cephalic (lateral) vein, the basilic (medial) vein, and the median vein. The superficial veins do not follow the course of the large arteries, presenting great interindividual variability in their course, confluence, and number. The deep veins (venae comitantes) accompany the radial and ulnar arteries (Figures 3 and 4). The superficial sensory portion of the musculocutaneous nerve (lateral cutaneous nerve of the forearm) has an intimate relationship with the cephalic vein along its course, best identified in the proximal portion of the forearm by HRUS^**(9)**^, as illustrated in Figure 8. The subcutaneous distal segment of the superficial branch of the radial nerve is in close proximity to the cephalic vein in the distal third of the forearm (Figure 4). The medial cutaneous nerve of the forearm is, in turn, near the basilic vein^**(9,10)**^, as depicted in Figure 8.

## CONCLUSION

For evaluating the peripheral nerves and muscles of the forearm, HRUS is an excellent method. The analysis of the relationships between vessels and nerves, which is relevant in intervention procedures, can be facilitated by the ancillary use of Doppler. Knowledge of the normal anatomy of the forearm improves the technical quality of the examinations, contributing to better diagnoses, as well as improving the safety and performance of interventional procedures.
